# Assessment of mitochondrial genomes for heterobranch gastropod phylogenetics

**DOI:** 10.1186/s12862-020-01728-y

**Published:** 2021-01-21

**Authors:** Rebecca M. Varney, Bastian Brenzinger, Manuel António E. Malaquias, Christopher P. Meyer, Michael Schrödl, Kevin M. Kocot

**Affiliations:** 1 Department of Biological Sciences, The University of Alabama, Campus Box 870344, Tuscaloosa, AL 35487 USA; 2 SNSB-Bavarian State Collection of Zoology, Münchhausenstr. 21, 81247 München, Germany; 3 Department of Natural History, University Museum of Bergen, University of Bergen, 5020 Bergen, Norway; 4 National Museum of Natural History, Smithsonian Institution, 10th St. & Constitution Ave. NW, Washington, D.C. 20560 USA; 5 BioGeoCenter LMU (Ludwig Maximillion University Munich), University of Munich, Biozentrum, Großhaderner Str. 2, 82152 Planegg-Martinsried, Germany; 6 Alabama Museum of Natural History, Campus Box 870344, Tuscaloosa, AL 35487 USA

**Keywords:** Heterobranchia, Gastropoda, Mitochondrial genome, Mitogenomic

## Abstract

**Background:**

Heterobranchia is a diverse clade of marine, freshwater, and terrestrial gastropod molluscs. It includes such disparate taxa as nudibranchs, sea hares, bubble snails, pulmonate land snails and slugs, and a number of (mostly small-bodied) poorly known snails and slugs collectively referred to as the “lower heterobranchs”. Evolutionary relationships within Heterobranchia have been challenging to resolve and the group has been subject to frequent and significant taxonomic revision. Mitochondrial (mt) genomes can be a useful molecular marker for phylogenetics but, to date, sequences have been available for only a relatively small subset of Heterobranchia.

**Results:**

To assess the utility of mitochondrial genomes for resolving evolutionary relationships within this clade, eleven new mt genomes were sequenced including representatives of several groups of “lower heterobranchs”. Maximum likelihood analyses of concatenated matrices of the thirteen protein coding genes found weak support for most higher-level relationships even after several taxa with extremely high rates of evolution were excluded. Bayesian inference with the CAT + GTR model resulted in a reconstruction that is much more consistent with the current understanding of heterobranch phylogeny. Notably, this analysis recovered Valvatoidea and Orbitestelloidea in a polytomy with a clade including all other heterobranchs, highlighting these taxa as important to understanding early heterobranch evolution. Also, dramatic gene rearrangements were detected within and between multiple clades. However, a single gene order is conserved across the majority of heterobranch clades.

**Conclusions:**

Analysis of mitochondrial genomes in a Bayesian framework with the site heterogeneous CAT + GTR model resulted in a topology largely consistent with the current understanding of heterobranch phylogeny. However, mitochondrial genomes appear to be too variable to serve as good phylogenetic markers for robustly resolving a number of deeper splits within this clade.

## Background

Mitochondrial genomes are popular molecular markers for animal phylogenetics: they are relatively easy to sequence, assemble, and annotate, typically have a moderate level of sequence conservation that facilitates phylogenetic comparisons among relatively distantly related taxa, and can have gene order rearrangements that are potentially phylogenetically informative. Phylogenetic analyses of mitochondrial genomes have clarified relationships within diverse groups of invertebrates such as crustaceans, echinoderms, sponges, hemichordates, and annelids, just to name a few [1–5]. However, the application of mitochondrial genomes to phylogenetics can be limited by differences in evolutionary rates (which can lead to long-branch attraction (LBA) artifacts and incomplete lineage sorting [6]. Mitochondrial genome-based studies of molluscan evolutionary relationships have been variable in success. Molluscan mitochondrial genomes exhibit wide variation in size, organization, and rate of evolution [7–9]. Mitochondrial genomes have substantially aided in clarification of relationships within clades such as Caudofoveata [10], Cephalopoda [11, 12], and some gastropod clades (e.g., [13–15]), but have had limited success at resolving relationships within other molluscan clades (e.g., [16]) and resolving deep molluscan phylogeny [17, 18].

Heterobranchia is a species-rich clade of gastropod molluscs that encompasses a wide diversity of snails and slugs that occupy marine, freshwater, and terrestrial habitats [19, 20]. Heterobranchs are thought to have diverged from other gastropods approximately 380 million years ago (mya; [21, 22]). Almost every clade within Heterobranchia has been subject to significant and continued taxonomic revision. The name Heterobranchia was coined by Burmeister (1837), but it is most commonly attributed to Gray (1840) who used it to unite Opisthobranchia (e.g., sea slugs) and Pulmonata (e.g., land snails). This group was later renamed Euthyneura to reflect the secondarily detorted arrangement of the cerebrovisceral commisures [23], but Heterobranchia was redefined to include Euthyneura and a grouping of taxa that are generally referred to as the “lower Heterobranchia” or Allogastropoda [24, 25] including, at times, Pyramidelloidea, Architectonicoidea, Valvatoidea, Orbitestelloidea, Omalogyridae, Rissoellidae, Glacidorbidae, Tjaernoeiidae, Cimidae, Rhodopemorpha, and Murchisonellidae [21, 22, 24–30]. Opisthobranchia has since been demonstrated to be a non-monophyletic group as sea slug clades such as Sacoglossa and Acochlidia share a more recent common ancestor with the pulmonates than other sea slugs as do some “lower” heterobranchs like Pyramidelloidea and Glacidorbidae [8, 13, 22, 31, 32], reviewed by [19].

Phylogenetic analyses to date have been unable to robustly resolve most relationships among major heterobranch clades. However, most of these studies have been limited by taxon sampling. In particular, many of the “lower heterobranchs” were missing from most investigations of heterobranch phylogeny to date. These snails and slugs are thought to represent a critical group to understanding heterobranch evolution as it has been debated whether they form a clade that is sister to all remaining heterobranchs or a “basal” paraphyletic grade. Here, we sequenced mitochondrial genomes from 11 heterobranch taxa, including several so-called lower heterobranchs and select other understudied clades. These new data were analyzed in combination with publicly available heterobranch and outgroup mitochondrial genomes to investigate the utility of mitochondrial genomes for resolving higher-level heterobranch phylogeny, placement of the lower heterobranchs, and the evolution of heterobranch mitochondrial genome organization.

## Results

### Genome assemblies and data matrix

We sequenced genomic DNA libraries from eleven species of heterobranch gastropods and extracted mitochondrial sequences (Table 1, Additional file 1: Table S1). Despite high sequencing coverage, a single contiguous mitochondrial genome was recovered for only two of the eleven taxa. All of the newly sequenced mt genomes were incomplete to some degree, possibly due to difficulties in sequencing through secondary structures associated with the 16S rDNA (which was absent from or incomplete in several of our assemblies) and the control region, but most of the mitochondrial protein-coding genes were obtained for all species. Alignments of amino acid sequences were produced for the thirteen protein-coding genes obtained from the newly sequenced taxa and publicly available heterobranch mt genomes on NCBI (Additional file 1: Table S1). After trimming each alignment to remove ambiguously aligned positions, the concatenated data matrix totaled 4735 amino acid sites with 31.3% gaps across 104 taxa (17 outgroups and 87 heterobranchs).

**Table 1:** Mitochondrial genomes sequenced in the present study and associated sources of samples

Taxon	mt contig length (bp)	Protein coding genes (13)	tRNAs (22)	rRNAs (2)	Complete	Missing genes	Sample accession	Provenance
*Acochlidium fijiense*	14,098	13	20	2	Nearly	trnS2, trnK	ZSM Mol-20130988	Fiji, Viti Levu, Lami River, collected by M Schrödl & E Schwabe, August 2006
*Clione limacina*	14,599	12	17	1	Nearly	trnl, atp8, trnS1, trnD, rrnL, trnY, trnR	ZSM Mol-20081086	Antarctica, ANDEEP SYSTCO Expedition on R/V Polarstern, ANTXXIV/2 cruise station PS 71/038-04, collected by E Schwabe & H Flores, January 2008
*Ilbia ilbi*	13,944	12	21	2	Nearly	atp8	MRG10019-03	Shoreham Beach 18 March 2017, collected by A Falconer
*Microdiscula charopa*	12,965	13	21	2	Yes		MRG828-06	Dutton Way, Portland 2 March 2017, approx 2 m deep, collected by A Falconer
*Omalogyra atomus*	12,413	10	20	1	No	atp8, rrnS, trnY, nad4l, trnV	ZSM Mol-20142011	France, Pyrénées-Orientales, Banyuls-sur-Mer, from red algae in upper intertidal, collected by B Brenzinger & TP Neusser, July 2014
*Psilaxis radiatus*	12,154	12	15	1	No	atp8, trnN, trnS1, tncC, trnF, trnY, rrnL, trnQ, trnV	ZMBN 94175	Museum of Zoology at the University of Bergen
*Ringicula conformis*	14,017	12	22	2	Nearly	nad4l	None	Malta, off Ġnejna Bay, 31 May 2017
*Rissoella morrocayensis*	11,085	12	5	1	No	trnS1, rrnL, trnI, trnN, trnC, trnF, trnY, trnQ,	ZMBN 99933	Museum of Zoology at the University of Bergen
*Runcina ornata*	13,862	13	22	2	Yes		ZMBN 87949	Museum of Zoology at the University of Bergen
*Tylodina* cf*. corticalis*	14,614	13	21	1	Yes	trnK, rrnL	USNM 1442311	French Polynesia, Moorea, NW side Cook’s Bay, collected by C McKeon, G Paulay, J O’Donnell and C Meyer, 12 June 2006
*Valvata* cf*. cristata*	14,495	13	21	1	Nearly	trnR, rrnL	ZSM Mol-20170210	Germany, Munich, pond on ZSM grounds, collected by B Brenzinger, March 2017

Yes: genome circularizes via overlapping ends, missing genes likely missed by annotator

Nearly: all genes listed on a single scaffold, but scaffold does not contain a full mitochondrion

No: mitochondrion either exists on two discontinuous scaffolds or is missing multiple protein coding genes

### Maximum likelihood analyses

A partitioned maximum likelihood (ML) analysis of this data matrix using the best-fitting model for each gene (Additional file 2: Figure S1; see additional data on FigShare for more information) resulted in a tree with *Valvata cristata* (Valvatoidea) recovered as the sister taxon to a clade containing all other heterobranchs with successive branching of *Microdiscula charopa* (Orbitestelloidea), a clade composed of *Clione limacina* (Gymnosomata), *Psilaxis radiatus* (Architectonicoidea), *Omalogyra atomus* (Omalogyroidea), and *Rissoella morrocayensis* (Rissoelloidae), and then *Rhopalocaulis grandidieri* (Veronicelloidea), which was the sister taxon of all remaining Heterobranchia. All members of the clade containing *C. limacina*, *P. radiatus*, *O. atomus*, and *R. morrocayensis* exhibited extremely long branches relative to the other heterobranchs and it is well-established that Gymnosomata is nested within Euopisthobranchia. Thus, we strongly suspected that this clade was the result of long-branch attraction. This, combined with very low backbone support values, led us to re-run the analysis with the following unstable and long-branched taxa removed: *C. limacina, P. radiatus, O. atomus,* and *R. morrocayensis* (see Additional file 10: Table S3).

**Fig. 1:**
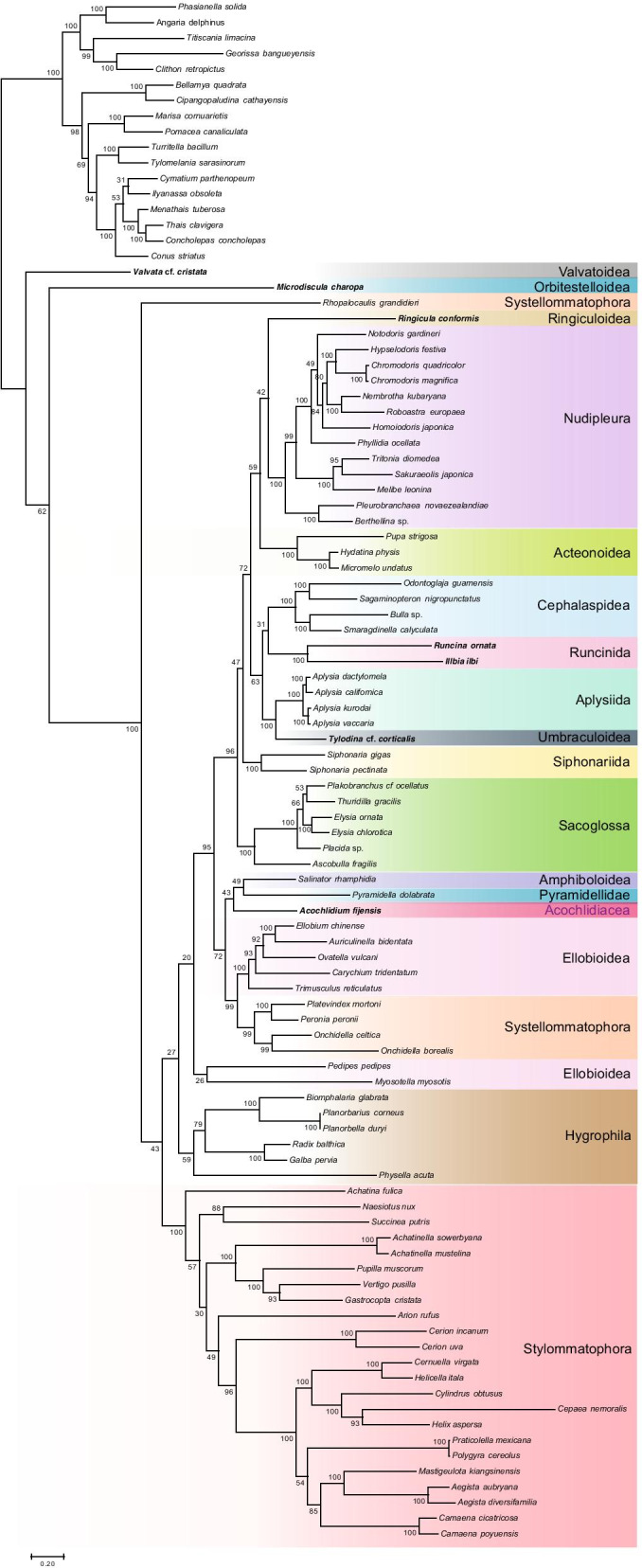
Maximum likelihood phylogeny of heterobranch gastropods based on the reduced set of taxa following removal of both unstable leaves flagged by RogueNaRok and the four longest-branched taxa. Taxa for which new sequences were collected are shown in bold. The data set was trimmed with TrimAL with default settings, partitioned by gene in RAxML, and the PROTGAMMAAUTO was used to select the best-fitting model for each partition. Bootstrap support values are presented at each node

The matrix with unstable and long-branched taxa removed totaled 4447 amino acid sites with 28.7% gaps across 99 taxa. In the resulting partitioned ML analysis using the best-fitting model for each gene (Fig. 1), *Valvata* was again recovered as the sister taxon to a clade composed of all other heterobranchs (bootstrap support, bs = 100) followed by the successive branching of *Microdiscula* (bs = 62) and *Rhopalocaulis* (bs = 100). Most major clades of Heterobranchia (Euthyneura sensu lato) were recovered with high support: Acteonoidea, Nudipleura, Cephalaspidea, Runcinida, Aplysiida, Siphonariida, Sacoglossa, and Stylommatophora were all recovered with 100% bootstrap support, and Systellommatophora with 99% bootstrap support. Of the family- and order-level taxa, Ellobioidea is the only one that was recovered as non-monophyletic, with *Pedipes pedipes* and *Myosotella myosotis* falling well outside of the clade containing the rest of Ellobioidea, albeit with very low support. Support for relationships among most higher-level heterobranch clades was generally weak and a number of higher-level groupings within Heterobranchia including Tectipleura, Euopisthobranchia, Panpulmonata, Eupulmonata, Systellommatophora, and Amphipulmonata (sensu [20]) were not monophyletic. However, Nudipleura (Nudibranchia + Pleurobranchomorpha) and a clade composed of Aplysiida + Umbraculoidea were recovered monophyletic with maximal support.

To explore the impact of different partitioning schemes on tree topology, and to determine whether other models better mitigated the long-branch attraction of *C. limacina, P. radiatus, O. atomus,* and *R. morrocayensis,* we ran several additional analyses. A partitioned analysis with a mixed model (LG + C60 + G + F) yielded a tree (Additional file 3: Figure S2) with the same clade of long-branched taxa as sister to all remaining heterobranchs except *Valvata* and *Microdiscula* and did not vary significantly in any other way from the original ML tree (Additional file 2: Figure S1). A ML analysis with Lanfear clustering (Additional file 4: Figure S3) and a fully partitioned ML analysis with resampling within partitions (Additional file 5: Figure S4) both produced similar trees to the initial partitioned ML analysis (Additional file 2: Figure S1), but with the two members of Ellobioidea that did not form a clade with the rest of Ellobioidea (*Myosotella* and *Pedipes*) falling outside Hygrophila and thus even farther from the remaining ellobioids. An analysis with an edge-unlinked model altered the relationships within the long-branched clade, with *O. atomus* and *P. radiatus* as sister to *R. morrocayensis* and *C. limacina,* and, while in previous analyses *R. morrocayensis* had a much longer branch than the other taxa in this clade, in the edge-unlinked tree, all four taxa had similarly elongated branches (Additional file 6: Figure S5). In this edge-unlinked analysis, the positions of Hygrophila and the pair of ellobiods were recovered with relationships similar to those of the initial partitioned ML tree (Additional file 2: Figure S1). We also analyzed the set of all taxa except *C. limacina* to assess whether removal of this single rapidly-evolving taxon would ‘release’ the other long-branched taxa, which are traditionally considered to be lower heterobranchs, from this putative LBA artifact. The other three long-branched taxa remained in the same location with long branches (Additional file 7: Figure S6).

### Bayesian inference

**Fig. 2:**
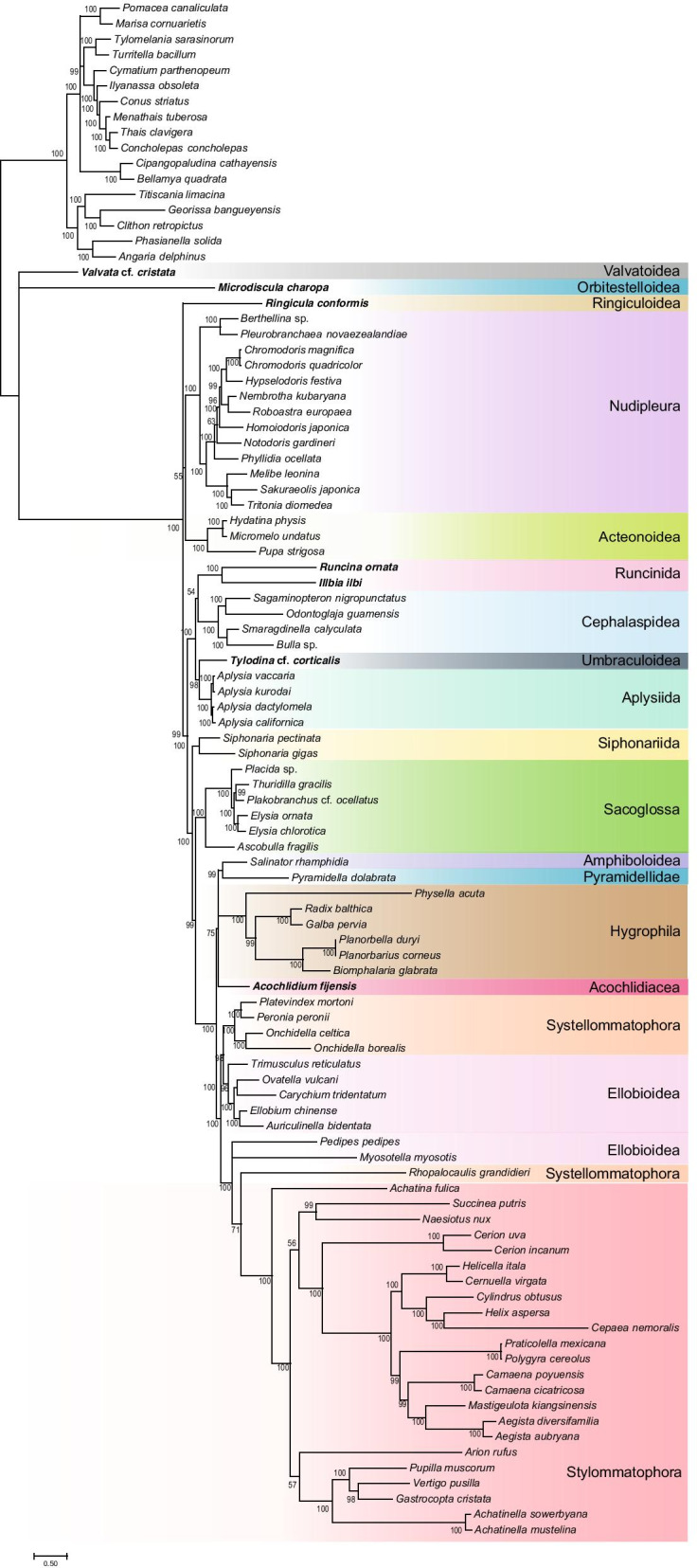
Bayesian inference phylogeny of Heterobranch molluscs based on the reduced set of taxa following removal of both unstable leaves flagged by RogueNaRok and the four longest-branched taxa. Taxa for which new sequences were collected in the present study are shown in bold. The data set was trimmed with BMGE and trees were generated in PhyloBayes with four chains using the CAT + GTR + Γ4 substitution model. Tree shown is the majority rule posterior consensus tree. Posterior probabilities are presented at each node

Because of poor support for most nodes of interest in our maximum likelihood analyses, we also performed a Bayesian inference with the CAT + GTR + G4 model on the same datasets, but only the analysis of the dataset with unstable and long-branched taxa removed reached convergence. Of the six chains that were run for over 60,000 generations, four converged according to the PhyloBayes bpcomp maxdiff criterion (maxdiff value = 0.29), yielding a tree with a topology that is much more consistent with the current understanding of heterobranch relationships (Fig. 2). *Valvata* and *Microdiscula* were recovered in a polytomy with a clade that comprised all other heterobranchs, which received maximal support. This clade consisted of a polytomy of Nudipleura + Acteonoidea, *Ringicula*, and the remaining heterobranchs. Nudipleura + Acteonoidea was weakly supported but Nudipleura again received maximal support.

The largest recovered subclade within Heterobranchia, Tectipleura, consisted of Euopisthobranchia (Cephalaspidea, Runcinida, Aplysiida, and Umbraculoidea) and Panpulmonata (Siphonariida, Sacoglossa, Hygrophila, Ellobioidea, Amphiboloidea, Systellommatophora, and Stylommatophora), which were recovered reciprocally monophyletic and both clades received maximal support. Within Euopisthobranchia, Cephalaspidea and Runcinida formed a (weakly supported) clade sister to a clade of Aplysiida + Umbraculoidea, the latter of which was strongly supported (posterior probability, pp = 0.98).

Within Panpulmonata, Siphonariida was recovered as the sister taxon to the rest of the clade (pp = 1) with Sacoglossa sister to all other taxa within that clade (pp = 0.96). The remaining panpulmonates formed two clades. One consisted of Stylommatophora, Systellommatophora, and Ellobioidea, although neither Systellommatophora nor Ellobioidea were recovered monophyletic. *Rhopalocaulis* (Systellommatophora) was recovered as the sister taxon of Stylommatophora (pp = 0.71) and this clade was recovered in a polytomy with the ellobioids *Pedipes* and *Myosotella* that was maximally supported. Sister to this polytomy was a moderately well-supported clade (pp = 0.98) in which the remainder of Systellommatophora (Onchidiidae) was sister to the remaining Ellobioidea. Sister to the Stylommatophora-Systellommatophora- Ellobioidea clade was a clade comprising Hygrophila (pp = 1.00), *Pyramidella dolabrata* (Pyramidelloidea), *Salinator rhamphidia* (Amphiboloidea), and *Acochlidium fijiense* (Acochlidia). *Salinator* and *Pyramidella* formed a well-supported clade (pp = 0.99) but otherwise, higher-level relationships in the Hygrophila-Pyramidelloidea-Amphiboloidea-Acochlidia clade were weakly supported.

### Mitochondrial gene order evolution

**Fig. 3:**
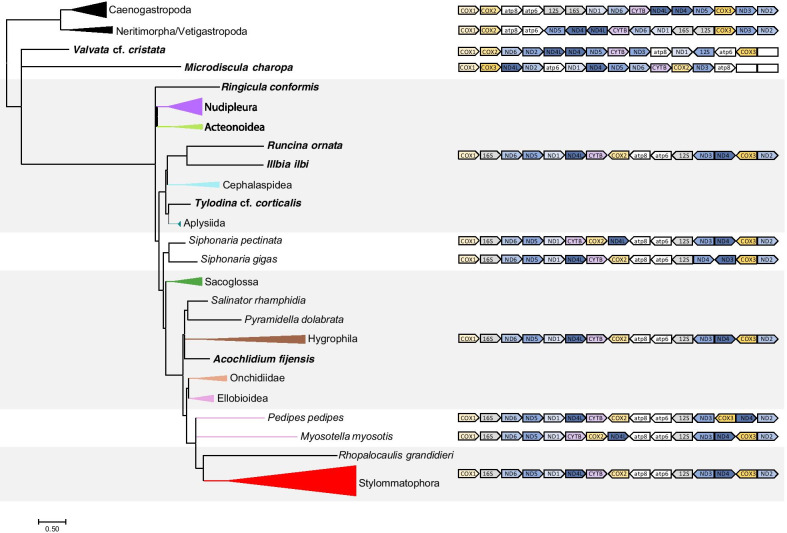
Presumed ancestral mitochondrial gene order based on a TreeRex analysis of each major clade of heterobranch gastropods. Grey boxes spanning multiple clades indicate the common Heterobranch gene order shared among most taxa. Empty white boxes represent missing genes from sequenced mitochondrial contigs. Tree topology is taken from the BI tree presented in Fig. 2

A somewhat diagnostic gene arrangement exists for heterobranchs relative to other gastropod clades, but many heterobranch taxa and subclades have differences in both gene organization and orientation in their mitochondrial genomes (Fig. 3). Caenogastropods encode all mitochondrial genes in the same orientation, while all members of the clade comprising Neritimorpha and Vetigastropoda share a single inversion of [12S rRNA, 16S rRNA, nad1, nad6, cytB, nad4L, nad4, nad5]. Across the diverse taxa used as outgroups in this study, no individual deviations in gene arrangement were found.

In contrast to this consistency, the taxa at the base of our heterobranch tree all have remarkably different mitochondrial gene arrangements from one another. Mitochondrial gene order within most of the “lower Heterobranchia” is variable: *Psilaxis radiatus* (Architectonicoidea)*, Omalogyra atomus* (Omalogyridae)*, Rissoella morrocayensis* (Rissoellidae)*,* and *Valvata* cf. *cristata* (Valvatidae) all have distinct gene orders from one another and from remaining heterobranchs, including changes in both order and orientation. *Microdiscula charopa* also has an entirely unique gene order.

In the remaining heterobranchs, the clade spanning Acteonoidea, Nudipleura, and the subclade including Runcinida, Cephalaspidea, Umbraculoidea, Aplysiida, Siphonariida, Sacoglossa, Amphiboloidea, Pyramidellidae, Hygrophila, Acochlidia, Systellommatophora, Ellobioidea, and Stylommatophora, a relatively stable mitochondrial gene order and orientation exists, referred to here as the common heterobranch gene order [cox1-16SrRNA-nad6-nad5-nad1-nad4L-ctyB-cox2-atp8-atp6-12SrRNA-nad3-nad4-cox3-nad2, with atp8-nad3 and cox3 both reversed in direction from cox1]. All members of Nudipleura examined adhere to this common gene order except *Hypselodoris festiva*, in which a single gene (nad4) changed position, and all members of Acteonoidea adhere to the same order as well. Variation exists within the Cephalaspidea, with a shared rearrangement of cytB, nad1, nad4L, and cox2 shared among three-fourths of its members, and *Sagaminopteron nigropunctatum* containing further rearrangements. Aplysiida adheres to the stable arrangement with the exception of *Aplysia kurodai*, in which the orientation of the 12S rRNA gene is reversed though its position remains the same.

Both representatives of Siphonariida have different internal mitochondrial gene rearrangements: *Siphonaria gigas* reversed the positions of nad4 and nad3, while *Siphonaria pectinata* inserts cox2 between nad4L and cytB. All sacoglossans shared a common gene order, as do *Pyramidella dolabrata* and *Acochlidium fijiensis*. The majority of Ellobioidea are consistent, excepting *Myosotella myosotis* and *Pedipes pedipes*, which have different single-gene transpositions than one another. Additionally, the mt genome of *P. pedipes* is expanded, with more intergenic space than other closely related taxa. Interestingly, these two taxa are those that fall together in a different part of the phylogeny than the remaining members of Ellobioidea, making this group paraphyletic. The clade comprising Hygrophila was strongly supported, and all members within it share the common heterobranch gene order except *Physella acuta*, which has a completely unique gene arrangement.

Within Stylommatophora, both members of genus *Achatinella* shared a single gene (cox2) moved to a different position relative to other members of the clade. Likewise, the taxa *Cylindrus obtusus, Cepaea nemoralis,* and *Cornu aspersum* (syn. *Helix aspersa*) all share a single gene (nad4) inserted at a different location in the mitochondrial genome. *Arion rufus* has the 12S rRNA placed prior to atp8-atp6 instead of after it, but all other members of Stylommatophora shared the common heterobranch gene order.

## Discussion

### Heterobranch phylogeny

We sequenced mitochondrial genomes from eleven heterobranch gastropods and investigated heterobranch evolutionary relationships using amino acid sequences from the thirteen mitochondrial protein-coding genes as well as the evolution of heterobranch mitochondrial genome organization. Mitochondrial genomes can be useful in molecular phylogenetics because of the functional constraint that should, in theory, lead to a relatively high degree of conservation across evolutionary time. This has been demonstrated in diverse groups of animals where mitochondrial genomes have served as useful markers for molecular phylogenetics [1–3, 5]. However, in other animal lineages, it has been demonstrated that mitochondrial genome evolutionary rate is too rapid to recover ancient radiations (e.g., [8, 17, 33]).

Our maximum likelihood-based analysis including all taxa failed to recover most recognized higher-level heterobranch clades but did recover a maximally supported clade of taxa with extremely long branches near the base of Heterobranchia. This clade includes taxa known to have brief lifespans, some of only a few months, which may correlate with a more rapid accumulation of genetic changes [34]. To combat this putative artifact of long-branch attraction, ML analyses of a dataset with long-branched and unstable taxa excluded were performed. Excluding these taxa resulted in a tree that exhibited an apparent mis-rooting within Heterobranchia relative to other studies (e.g., [22, 31, 32], reviewed by [19]) with Panpulmonata paraphyletic with respect to a clade of opisthobranchs. Support for most higher-level heterobranch clades was weak in both maximum likelihood analyses, although most order-level taxa were recovered monophyletic with strong support.

Clear long-branch attraction and weak support for deep relationships within Heterobranchia initially led us to conclude that mitochondrial genomes have little to no phylogenetic signal for deep nodes within Heterobranchia. Additional maximum likelihood analyses that attempted to account for differences in evolutionary rates between genes did not improve resolution of these deeper nodes. However, although mostly weakly supported, a number of previously hypothesized relationships were recovered in all of our maximum likelihood analyses. These include Euthyneura (e.g. all heterobranchs except Valvatoidea and Orbitestelloidea), Pyramidelloidea + Amphiboloidea, Nudipleura + *Ringicula* (Ringipleura), Ringipleura + Acteonoidea (not considering Rissoelloidae), Aplysiida + Umbraculoidea (not considering Gymnosomata and Thecosomata), and Cephalaspidea + Runcinida [22, 31, 32, 35–38].

In order to determine if selecting a model that better accounts for site-specific rate heterogeneity could help improve resolution, we conducted a Bayesian inference using the site heterogeneous CAT + GTR + G4 model. This analysis resulted in a topology that is much more consistent with other studies examining heterobranch evolutionary relationships to date. Again, we recovered Euthyneura to the exclusion of Valvatoidea and Orbitestelloidea with maximal support. Whereas our maximum likelihood analyses recovered Valvatoidea as the sister taxon to all other heterobranchs with moderate to weak support, our Bayesian inference recovered these two “lower heterobranchs” in a polytomy with the rest of Heterobranchia. This is in concordance with previous Sanger-sequencing based approaches [20, 29] but neither clade was so far sampled by phylogenomics [35] or mitogenomics [15]. Valvatoidea (= Ectobranchia) is a group of minute freshwater and marine snails with discoidal to ovoid shells. Haszprunar et al. regarded Valvatoidea as the earliest-branching heterobranch clade based on their broad, rhipidoglossate radula and unusual ectobranch gill [39]. This phylogenetic position was favored by Brenzinger et al. because a clade of all heterobranchs except Valvatoidea is supported by the presence of ciliated strips in the mantle cavity [40]. Sperm ultrastrucure is also consistent with their placement among the lower heterobranchs [41]. Orbitestelloidea was considered to belong to Valvatoidea in the past. Our Bayesian analysis produced a polytomy containing these taxa, but all our maximum likelihood analyses separated these taxa from one another with Valvatoidea sister to all other heterobranchs, as consistent with the most recent classification [27]. The fossil record also coincides with a greater age of “lower” heterobranchs (possibly present in mid-Paleozoic) vs. Euthyneura (diversifying in the Jurassic) [42–44], although unequivocal fossils of Valvatoidea and Orbitestelloidea—with minute, fragile and often inconspicuous shells—are much younger (Cretaceous to Eocene) (see [42, 43]). Architectonicoidea is another candidate for an old group judging from the presence of fossils in the Triassic [45]. Unfortunately, most of the other lower heterobranchs we sampled—*O. atomus* (Omalogyroidea), *Psilaxis radiatus* (Architectonicoidea), and *Rissoella morrocayensis* (Rissoelloidae)—exhibited extremely long branches and the Bayesian inference including these taxa (as well as an analysis including these taxa but excluding *C. limacina*; data not shown) failed to converge.

Our Bayesian inference recovered Pyramidelloidea + Amphiboloidea and Aplysiida + Umbraculoidea with strong support (pp ≥ 0.98). This analysis also recovered a number of other heterobranch clades that have been identified in other studies but were not recovered in the maximum likelihood analysis of this dataset. Most notably among these is Panpulmonata. We recovered Siphonariida as the sister taxon of the remaining panpulmonates followed by Sacoglossa as the sister taxon to all other panpulmonates after that, all with strong support (pp ≥ 0.99). Interestingly, support for the relative placement of these two clades has been weak in most studies with the relevant taxon sampling to date (but see [31]). Our results are inconsistent with most studies to date, which have recovered these two taxa as a clade [22, 38] or with Sacoglossa, not Siphonariida, sister to the rest of Panpulmonata [31, 32], reviewed by [20, 46].

Although *Ringicula* was previously recovered as the sister taxon of Nudipleura [32], which we recovered here in our maximum likelihood analyses, this relationship was not supported in our Bayesian inference. Instead, Nudipleura was recovered as the sister taxon of Acteonoidea, but this clade was weakly supported. *Ringicula* was recovered in a polytomy with this weakly supported Nudipleura-Acteonoidea clade (and a strongly supported clade consisting of all other heterobranchs), so although we find no support for the Ringipleura hypothesis in this analysis, our Bayesian inference results are not incompatible with Ringipleura.

All of our analyses failed to recover Ellobioidea as a monophyletic group. A previous analysis that included some of the Ellobioidea mitochondrial genomes analyzed herein, including those of the two taxa that were recovered separately from the rest of Ellobioidea in our analyses (*Pedipes pedipes* and *Myosotella myosotis*), also failed to recover a monophyletic Ellobioidea [47]*.* However, Dayrat et al. and Romero et al. sampled both of these species and recovered them as nested within Ellobioidea (although Dayrat et al. also recovered *Trimusculus* and *Otininae* within Ellobioidea) [48, 49].

### Evolution of heterobranch mitochondrial gene organization

Long-branch attraction, as discussed above, is likely responsible for the recovery of *C. limacina* in a clade with the “lower heterobranchs” *O. atomus, P. radiatus,* and *R. morrocayensis.* Often there is a correlation between a high rate of genome evolution and genome rearrangements [50]; *O. atomus* and members of the genus *Rissoella* are known to have short life cycles [20, 51]. *C. limacina* has a completely unique gene order, so it is possible that some sequence differences may be a result of rearrangement and these in turn contributed to the misplacement of this taxon. Within Ellobioidea, the two members that are consistently recovered apart from the rest (*Myosotella myosotis* and *Pedipes pedipes*) both contain single gene transpositions (though differing from one another).

However, this correlation does not hold for other isolated members of clades with extreme gene order rearrangements. For example, *Physella acuta* has mitochondrial gene reordering so extensive that a minimum of five independent gene rearrangements are necessary to account for the difference between it and the remaining members of Hygrophila [52]. Despite this, *P. acuta* still forms a clade with the rest of Hygrophila with very high support in all analyses. Likewise, *Sagaminopteron nigropunctatus* forms a clade with the other cephalaspids with very high support despite differing dramatically in gene arrangement from the other three members included in the analysis, and the relationships among these taxa are supported by recent transcriptome-based analyses [53]. The variable relationship of evolutionary rate of gene sequences and mitochondrial gene rearrangement could be interesting to investigate in future studies.

The shared gene arrangement among the majority of heterobranch taxa suggests this common gene order emerged prior to the common ancestor of Nudipleura and remaining taxa. However, most taxa previously identified as “lower heterobranchs,” as well as the additional taxa recovered at the base of the heterobranch tree in our analyses, have unique mitochondrial gene arrangements relative to all other gastropods. The differences among these taxa and between them and the main clade of heterobranchs cannot be explained with stepwise changes, but instead suggest multiple independent inversions and transpositions and may be due to a combination of long evolutionary trajectories (since the mid-Paleozoic) [54] and derived ecologies and lifestyles in many subgroups, including the commonly observed abbreviation and modification of life cycles by multiple evolution of paedomorphic groups. Our results indicate that mitochondrial genome organization started to deviate considerably from the ancestral molluscan arrangement first at the origin of Heterobranchia and later, even more so, at the origin of Euthyneura.

## Conclusions

Here, we sequenced 11 new heterobranch mitochondrial genomes including several “lower heterobranchs”. These new data were analyzed in combination with publicly available heterobranch and outgroup mitochondrial genomes using maximum likelihood and Bayesian inference. Results of maximum likelihood analyses with site-homogeneous models indicated that even with the exclusion of exceptionally rapidly evolving taxa, mitochondrial genomes have limited utility for resolving most higher-level heterobranch relationships. However, Bayesian inference using the site-heterogeneous CAT + GTR + G model recovered most recognized higher-level heterobranch clades including Tectipleura, Euopisthobranchia, and Panpulmonata. Unfortunately, most of the lower heterobranch taxa that we aimed to place in a phylogenetic context exhibited extremely fast rates of evolution. Relationships within most heterobranch order-level clades that were broadly sampled (e.g., Nudipleura, Aplysiida, Sacoglossa, Hygrophila, Stylommatophora) were well-resolved and strongly supported. Despite the relatively rapid rate of nucleotide evolution in heterobranch mitochondrial genomes, gene order was found to be largely conserved across the group. Taken together, these results provide support for several hypothesized heterobranch clades and highlight the non-euthyneuran clades Valvata and Orbitestelloidea as interesting and important taxa to study with respect to understanding early heterobranch evolution. However, a lack of resolution and poor support for a number of deeper nodes within Heterobranchia highlight limitations of mitogenomic data for deep phylogeny, especially for rapidly evolving taxa like the long-branched lower heterobranchs, and reveals the surprising degree of heterogeneity within even closely related molluscan taxa that may in part be responsible for these limitations.

## Methods

### DNA extraction, library preparation, and sequencing

DNA was extracted from specimens obtained from various sources (Table 1) using the Omega Bio-tek EZNA MicroElute Genomic DNA Kit (Omega Bio-tek, Norcross, GA) or with a MO-BIO Powermax Soil DNA Isolation Kit. As most of the newly sequenced taxa were small-bodied, in most cases entire specimens were placed directly into lysis buffer, and if size permitted, were ground with a sterile pestle prior to digestion to break open shells. DNA concentration was measured using a Qubit 4 Fluorometer (Thermo Fisher Scientific, Waltham, MA) with the ds DNA HS kit. Samples that yielded too little DNA for library preparation (*Rissoella morrocayensis* and *Omalogyra atomus*) were amplified with multiple strand displacement amplification using the Illustra Single Cell GenomiPhi DNA Amplification Kit (GE Healthcare, Chicago, IL). Dual-indexed sequencing libraries were prepared with the Illumina Nextera Kit (Illumina, San Diego, CA). Library size was assessed via agarose gel following a test PCR (run with provided Illumina primers 1.1 and 2.1, run 95 °C for 10 min followed by 40 cycles of [95° for 10 s, 60° for 30 s]). Pooled libraries were sequenced with a 2 × 100 bp paired-end TruSeq 3000/4000 SBS kit on an Illumina HiSeq4000 (Macrogen, South Korea) using 1/24 lane each.

### Assembly and annotation

De novo assemblies of reads were initially carried out with Spades 3.14.0 [55]. In the case of *O. atomus*, the longest mitochondrial genome contig produced by Spades was missing several mitochondrial genes and Ray 2.2.0 [56] was used for assembly. Mitochondrial genomes were identified by creating a BLAST database from each set of assembled scaffolds and querying that database with the complete mitochondrion of *Galba pervia* (NCBI NC_018536.1) via BLASTN with an e-value cutoff of 1e-4. The longest BLAST hits were annotated with the MITOS2 web server with default parameters and the invertebrate mitochondrial genetic code (5) [57].

### Data set construction

Coding sequences of the 13 mitochondrial protein-coding genes (*cox1, cox2, cox3, atp6, atp8, nad1, nad2, nad3, nad4, nad4L, nad5, nad6, cob*) were extracted from the newly sequenced and annotated mitochondrial genomes, as well as those publicly available on NCBI (see Additional file 1: Table S1). Single-gene alignments were simultaneously produced for DNA and amino acid sequences with MACSE v1.2 [7] using the invertebrate genetic code (5). Alignments were trimmed with trimAL with default settings [58]. Trimmed alignments were checked manually in MEGA 10.0.4 [59] and corrected by hand if translations were initially out of frame. FASconCAT [60] was used to assemble the concatenated amino acid supermatrix file. In response to difficulties with long-branched taxa (Additional file 2: Figure S1) and in keeping with recent recommendations to improve phylogenetic analysis [61], the alignment was also trimmed with BMGE [62] trimming with default settings. The BMGE matrix was used for subsequent analyses. All data matrices are available online via FigShare.

### Maximum likelihood analyses

An initial maximum likelihood analysis (Supplemental Figure S1) was conducted on the initial TrimAL-trimmed (with default settings), partitioned-by-gene supermatrix using RAxML v8.2.4 [63] with the PROTGAMMAUTO model, which automatically selects the best-fitting model for each partition, rapid bootstrapping, and selection of the best-scoring maximum likelihood tree in one run. The number of bootstrap replicates was determined by the majority-rule consensus criterion (autoMRE).

Leaf stability was assessed with RogueNaRok [64] using the majority rule consensus criterion. Four taxa (*R. grandideri, P. pedipes, P. acuta,* and *M. myosotis*) had a leaf stability difference of < 0.75 and were considered to be unstable by RogueNaRok (Additional file 8: Table S2). These taxa, along with the very long-branched taxa *C. limacina, P. radiatus*, *O. atomus*, and *R. morrocayensis* were removed and the remaining sequences for each gene were re-aligned, trimmed, and concatenated before reanalysis in RAxML as described above.

To attempt to combat the apparent long branch attraction among *C. limacina, P. radiatus*, *O. atomus*, and *R. morrocayensis,* trees were also produced for the BMGE-trimmed matrix with a number of different models and/or analysis settings. We performed a series of ML analyses in IQ-TREE 2 [65] with 1000 rapid bootstraps employing different models and partitioning schemes including (1) the BMGE-trimmed dataset partitioned by gene with a partitioned mixed model (LG + C60 + G + F) and the best tree from RAxML provided as a starting tree (Additional file 3: Figure S2); (2) the same BMGE-trimmed dataset partitioned by gene and using Lanfear clustering to select optimal partitioning [66], resulting in 5 partitions with different models (Additional file 4: Figure S3); (3) a fully partitioned analysis of this matrix where PartitionFinder independently selected the best model for each gene with the –GENESITE correction to resample partitions and then sites within partitions [67] (Additional file 5: Figure S4); (4) an analysis of this matrix with an edge-unlinked model to better account for heterotachy (GHOST) [68] (Additional file 6: Figure S5). We also ran a RAxML analysis on the original TrimAL-trimmed dataset but with *C. limacina* removed (Additional file 7: Figure S6. In all IQ-TREE 2 and RAxML trees, the clade of four (or three, in the last analysis) long-branched taxa persisted, and the overall tree topology did not change.

### Bayesian analysis

Bayesian trees were generated with PhyloBayes-MPI v1.6 [69] with four chains and the CAT + GTR + Γ4 substitution model. Two analyses were attempted based on the BMGE-trimmed matrix: (1) an analysis sampling all taxa; and (2) an analysis excluding the taxa flagged as unstable in the initial maximum likelihood tree (*C. limacina, P. radiatus, O. atomus,* and *R. morrocayensis*).

### Mitochondrial gene order

In light of the apparent heterogeneity in mitochondrial gene sequences within and between clades, we examined gene order across major groups with TreeREx v1.85 [70]. The heterogeneity within several groups made it impossible to visualize all at once (Additional file 8: Figure S7), so nodes of major clades were collapsed and the inferred ancestral gene arrangement for each clade diagrammed again with TreeREx (Additional file 11: Figure S8). Syntenic blocks were visualized with Geneious R11 (Additional file 10: Table S3).

## Supplementary Information

**Additional file 1: Table S1.** Data downloaded from NCBI used in the present study.

**Additional file 2: Figure S1.** Maximum likelihood phylogeny of heterobranch gastropods based on the full set of available heterobranch mitochondrial genomes (including long-branched taxa). The data set was partioned by gene, trimmed with TrimAL with default settings, and analyzed in RAxML with the PROTGAMMAAUTO setting to select the best-fitting model for each partition. Bootstrap support values are presented at each node.

**Additional file 3: Figure S2.** Maximum likelihood phylogeny of heterobranch gastropods based on the full set of available heterobranch mitochondrial genomes (including long-branched taxa). The data set was partitioned by gene, trimmed with BMGE, and analyzed in IQ-TREE 2 with the LG + C60 + G + F mixed model. Bootstrap support values are presented at each node.

**Additional file 4: Figure S3.** Maximum likelihood phylogeny of heterobranch gastropods based on the full set of available heterobranch mitochondrial genomes (including long-branched taxa). The data set was partitioned by gene, trimmed with BMGE, and greedy Lanfear clustering was applied in IQ-TREE 2 to determine the optimal partitioning scheme. Five partitions with independent models were applied. Bootstrap support values are presented at each node.

**Additional file 5: Figure S4.** Maximum likelihood phylogeny of heterobranch gastropods based on the full set of available heterobranch mitochondrial genomes (including long-branched taxa). The data set was partitioned by gene, trimmed with BMGE, and each partition was allowed to select its own optimal model via ModelFinder implemented in IQ-TREE 2. The analysis was run with the –GENESITE correction to facilitate resampling first within partition and then within sites. Bootstrap support values are presented at each node.

**Additional file 6: Figure S5.** Maximum likelihood phylogeny of heterobranch gastropods based on the full set of available heterobranch mitochondrial genomes (including long-branched taxa). The data set was trimmed with BMGE, concatenated into a supermatrix, and analyzed with an edge-unlinked model to better account for heterotachy (GHOST). The analysis was run in IQ-TREE 2 with the –GENESITE correction to facilitate resampling first within partition and then within sites. Bootstrap support values are presented at each node.

**Additional file 7: Figure S6.** Maximum likelihood phylogeny of heterobranch gastropods based on the full set of available heterobranch mitochondrial genomes (including long-branched taxa) except for *C. limacina*. The data set was partitioned, trimmed with TrimAL with default settings, and concatenated into a supermatrix, and run in RAxML with -PROTGAMMAAUTO setting to select the best-fitting model.

**Additional file 8: Figure S7.** TreeRex output of a rearrangement analysis highlighting the multiple inversions and transpositions across heterobranch mitochondrial genomes. Rearrangements shown on branches are delineated as T for transposition and TDL for tandem-duplication-random-loss events. Nodes colored green as consistently reconstructed, red reconstructed with the fallback method (where P0 indicates the chosen solution is not better than other possible solutions).

**Additional file 9: Table S2.** RogueNaRok leaf instability indices, run with the tree from Figure 1. Lsi_42_max represents the maximum leaf instability across four possible quartets, lsi_42_ent the entropy between the two most different quartets, and Lsi_42_dif the leaf stability differences between the two most common quartets.

**Additional file 10: Figure S8:** TreeRex output of a subset of representative mitochondrial genomes from heterobranch gastropods to showcase more general patterns in rearrangements between major clades. Rearrangements shown on branches are delineated as T for transposition and TDL for tandem-duplication-random-loss events. Nodes colored green as consistently reconstructed, red reconstructed with the fallback method (where P0 indicates the chosen solution is not better than other possible solutions).

**Additional file 11: Table S3.** Mitochondrial gene orders in all taxa from the present study, including outgroups, with < and > indicating directionality and orange boxes indicating possible locations of gene rearrangements.

## Data Availability

The raw Illumina FASTQ reads generated in this study are available via NCBI SRA under BioProject ID PRJNA626646. Mitochondrial sequences and annotations are also available under NCBI BioProject PRJNA626646. Single gene alignments, data matrices, and tree files are available viaFigShare: https://figshare.com/projects/Assessment_of_mitochondrial_genomes_for_heterobranch_gastropod_phylogenetics/80765.

## Abbreviation

mt: Mitochondrial
