# Supplementary material for: Assessment of mitochondrial genomes for heterobranch gastropod phylogenetics
Source: BMC Ecol Evol. 2021 Jan 21;21:6. doi: 10.1186/s12862-020-01728-y (PMC7853304; doi:10.1186/s12862-020-01728-y)
Supplement: Supplementary file 1 — Additional file 1: Table S1. Data downloaded from NCBI used in the present study. [file 12862_2020_1728_MOESM1_ESM.docx]

Table 1: Mitochondrial genomes downloaded from NCBI (including outgroups).

| **Clade** | **Species** | **Accession Number** |
| --- | --- | --- |
| Caenogastropoda | Ballamya quadrata | NC_031850.1 |
| Caenogastropoda | Cipangopaludina cathayensis | NC_025577.1 |
| Caenogastropoda | Concholepas concholepas | JQ446041.1 |
| Caenogastropoda | Conus striatus | KX156937.1 |
| Caenogastropoda | Cymatium parthenopeum | EU827200.1 |
| Caenogastropoda | Ilyanassa obsoleta | DQ238598.1 |
| Caenogastropoda | Marisa cornuarietis | NC_025334.1 |
| Caenogastropoda | Menathais tuberosa | KU747972.1 |
| Caenogastropoda | Pomacea canaliculata | KU052865.1 |
| Caenogastropoda | Thais clavigera | DQ159954.1 |
| Caenogastropoda | Turritella bacillum | KU221394.1 |
| Caenogastropoda | Tylomelania sarasinorum | KU878411.1 |
| Neritimorpha | Clithon_retropictus | NC_031893.1 |
| Neritimorpha | Titiscania_limacina | KU342669.1 |
| Vetigastropoda | Angaria_delphinus | NC_031860.1 |
| Vetigastropoda | Phasianella_solida | NC_028709.1 |
| Acteonoidea | Hydatina_physis | DQ991932.1 |
| Acteonoidea | Micromelo_undatus | DQ991933.1 |
| Acteonoidea | Pupa_strigosa | AB028237.1 |
| Amphiboloidea | Salinator_rhamphidia | JN620539.1 |
| Anaspidea | Aplysia_californica | AY569552.1 |
| Anaspidea | Aplysia_dactylomela | DQ991927.1 |
| Anaspidea | Aplysia_kurodai | KF148053.1 |
| Anaspidea | Aplysia_vaccaria | DQ991928.1 |
| Cephalasipdea | Bulla_sp. | DQ991930.1 |
| Cephalasipdea | Odontoglaja_guamensis | DQ991935.1 |
| Cephalasipdea | Sagaminopteron_nigropunctatus | DQ991937.1 |
| Cephalasipdea | Smaragdinella_calyculata | DQ991938.1 |
| Ellobioidea | Auriculinella_bidentata | JN606066.1 |
| Ellobioidea | Carychium tridentatum | KT696545.1 |
| Ellobioidea | Ellobium chinense | NC_034292.1 |
| Ellobioidea | Myosotella_myosotis | AY345053.2 |
| Ellobioidea | Myosotella_myosotis | JN606067.1 |
| Ellobioidea | Ovatella_vulcani | JN615139.1 |
| Ellobioidea | Pedipes_pedipes | JN615140.1 |
| Hygrophila | Biomphalaria_glabrata | AY380567.1 |
| Hygrophila | Galba_pervia | JN564796.1 |
| Hygrophila | Physella_acuta | JQ390525.1 |
| Hygrophila | Planorbarius_corneus | KP279639.1 |
| Hygrophila | Radix_balthica | KP098541.1 |
| Nudibranchia | Chromodoris_magnifica | DQ991931.1 |
| Nudibranchia | Chromodoris_quadricolor | KU317089.1 |
| Nudibranchia | Hypselodoris_festiva | KU365323.1 |
| Nudibranchia | Melibe_leonina | KP764764.1 |
| Nudibranchia | Notodoris_gardineri | DQ991934.1 |
| Nudibranchia | Phyllidia_ocellata | KU351090.1 |
| Nudibranchia | Roboastra_europaea | AY083457.1 |
| Nudibranchia | Tritonia_diomedea | KP764765.1 |
| Nudipleura | Homoiodoris japonica | NC_034006.1 |
| Nudipleura | Nembrotha kubaryana | KY131978.1 |
| Nudipleura | Sakuraeolis japonica | NC_033968.1 |
| Onchidiidae | Onchidella_borealis | DQ991936.1 |
| Onchidiidae | Onchidella_celtica | AY345048.2 |
| Onchidiidae | Peronia_peronii | JN619346.1 |
| Onchidiidae | Platevindex_mortoni | GU475132.1 |
| Planorboidea | Planorbella duryi | KY514384.1 |
| Pleurobranchomorpha | Berthellina_sp. | DQ991929.1 |
| Pleurobranchomorpha | Pleurobranchaea_novaezealandiae | KU365727.1 |
| Pleurobranchomorpha | Pleurobranchaea_sp. | KU365728.1 |
| Pyramidellidae | Pyramidella_dolabrata | AY345054.2 |
| Sacoglossa | Ascobulla_fragilis | AY345022.2 |
| Sacoglossa | Elysia_chlorotica | EU599581.1 |
| Sacoglossa | Elysia_ornata | KU365324.1 |
| Sacoglossa | Placida_sp. | KC171014.1 |
| Sacoglossa | Plankobranchus cf ocellatus | KX853083.1 |
| Sacoglossa | Thuridilla_gracilis | DQ991939.1 |
| Siphonariidae | Siphonaria_gigas | JN627205.1 |
| Siphonariidae | Siphonaria_pectinata | AY345049.2 |
| Stylommatophora | Achatina_fulica | KJ744205.1 |
| Stylommatophora | Achatinella mustelina | NC_030190.1 |
| Stylommatophora | Achatinella_sowerbyana | KX356680.1 |
| Stylommatophora | Aegista aubryana | NC_029419.1 |
| Stylommatophora | Aegista_diversifamilia | KR002567.1 |
| Stylommatophora | Arion rufus | KT626607.1 |
| Stylommatophora | Camaena poyuensis | KT001074.1 |
| Stylommatophora | Camaena_cicatricosa | KM365408.1 |
| Stylommatophora | Cepaea_nemoralis | U23045.1 |
| Stylommatophora | Cerion uva | NC_034226.1 |
| Stylommatophora | Cerion_incanum | KM365085.1 |
| Stylommatophora | Cernuella_virgata | KR736333.1 |
| Stylommatophora | Cylindrus_obtusus | JN107636.1 |
| Stylommatophora | Gastrocopta_cristata | KC185403.1 |
| Stylommatophora | Helicella itala | KT696546.1 |
| Stylommatophora | Helix_aspersa | JQ417194.1 |
| Stylommatophora | Mastigeulota_kiangsinensis | KM083123.1 |
| Stylommatophora | Naesiotus_nux | KT821554.1 |
| Stylommatophora | Polygyra cereolus | NC_032036.1 |
| Stylommatophora | Praticolella mexicana | NC_032079.1 |
| Stylommatophora | Pupilla_muscorum | KC185404.1 |
| Stylommatophora | Succinea_putris | JN627206.1 |
| Stylommatophora | Vertigo_pusilla | KC185405.1 |
| Systellomatophora | Rhopalocaulis_grandidieri | JN619347.1 |
| Trimusculoidea | Trimusculus_reticulatus | JN632509.1 |
