# Supplementary figures and images for: Assessment of mitochondrial genomes for heterobranch gastropod phylogenetics
Source: BMC Ecol Evol. 2021 Jan 21;21:6. doi: 10.1186/s12862-020-01728-y (PMC7853304; doi:10.1186/s12862-020-01728-y)

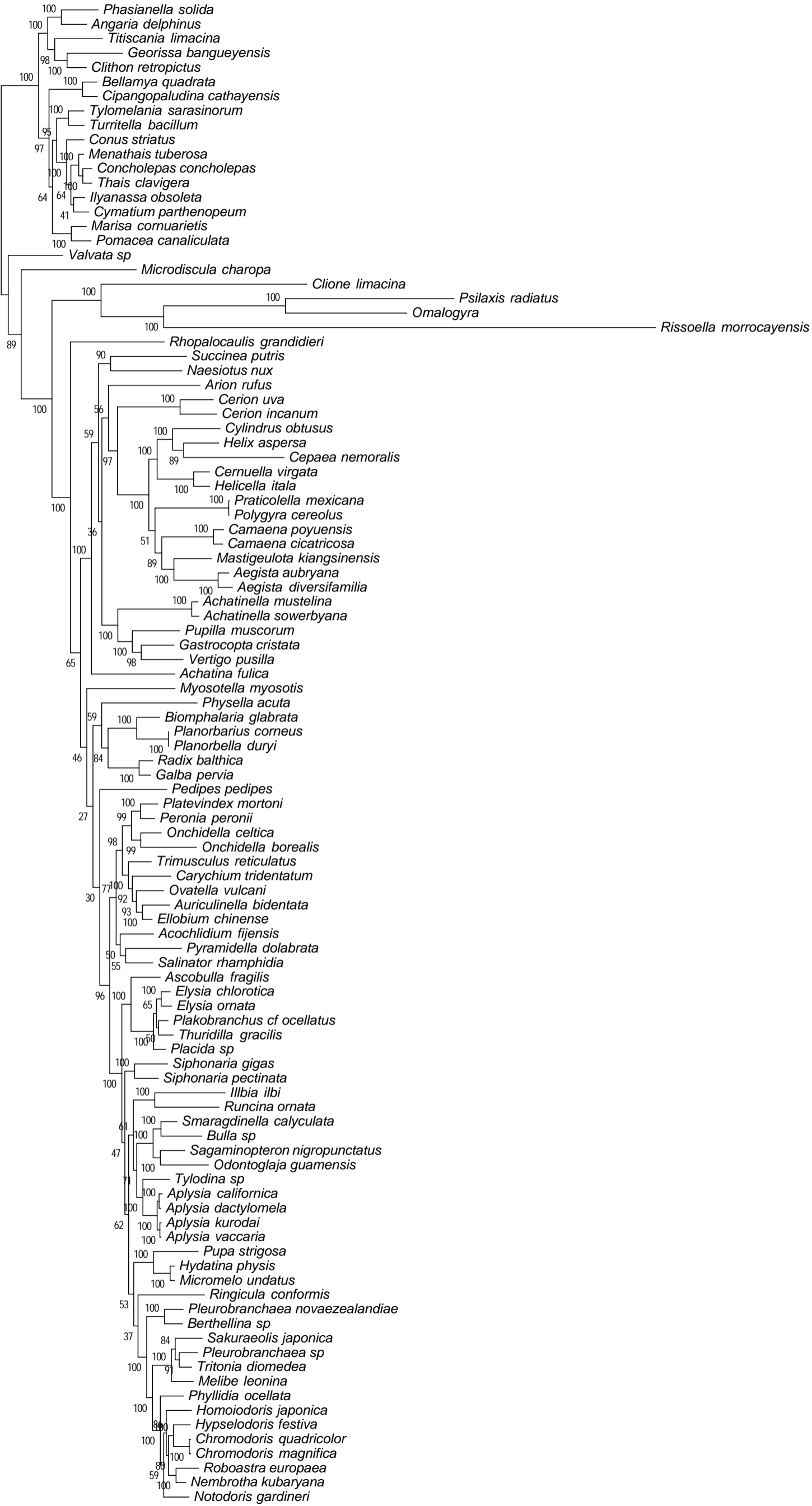

0.20

Supplement: Supplementary file 2 — Additional file 2: Figure S1. Maximum likelihood phylogeny of heterobranch gastropods based on the full set of available heterobranch mitochondrial genomes (including long-branched taxa). The data set was partioned by gene, trimmed with TrimAL with default settings, and analyzed in RAxML with the PROTGAMMAAUTO setting to select the best-fitting model for each partition. Bootstrap support values are presented at each node. [file 12862_2020_1728_MOESM2_ESM.pdf]

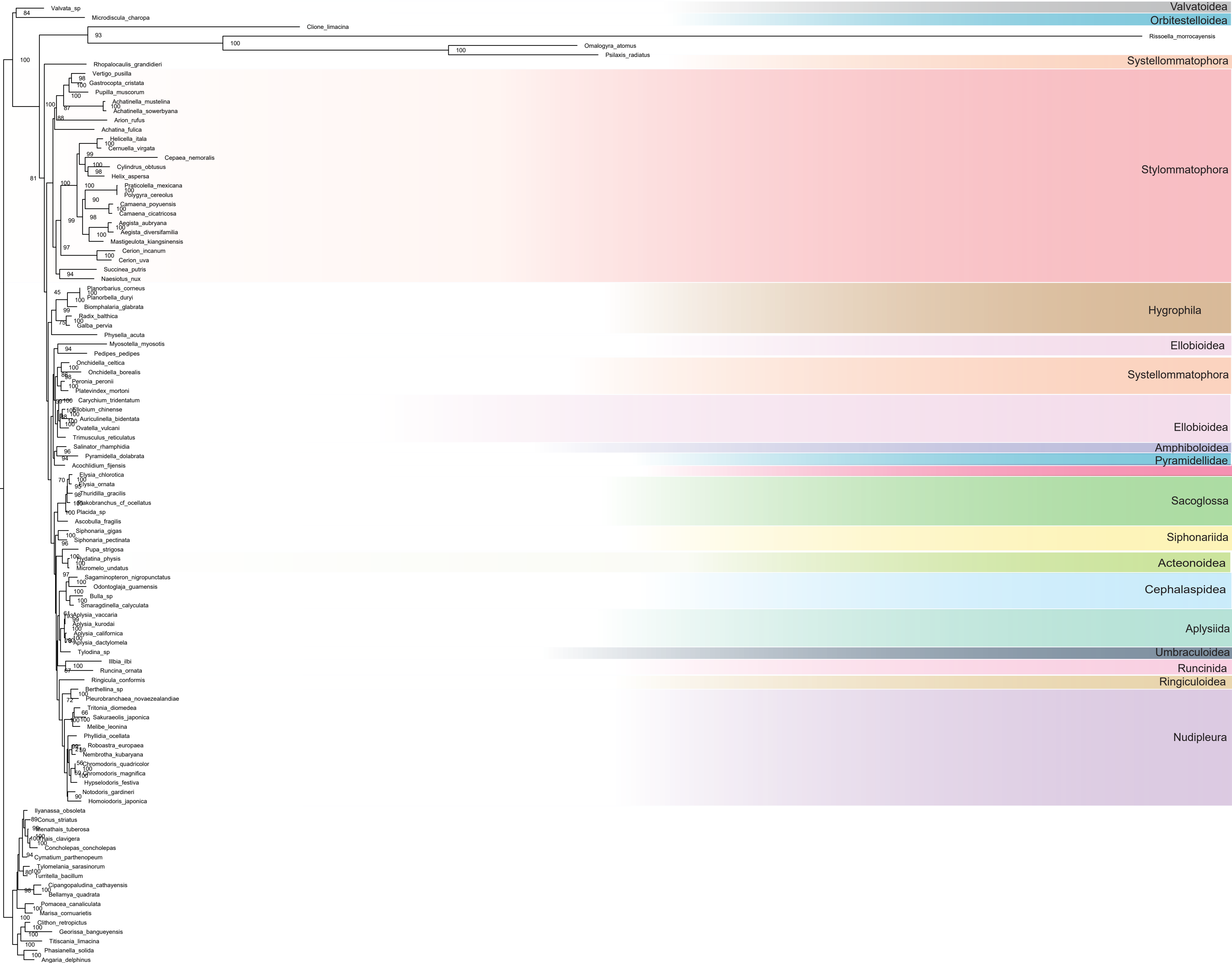

Supplement: Supplementary file 3 — Additional file 3: Figure S2. Maximum likelihood phylogeny of heterobranch gastropods based on the full set of available heterobranch mitochondrial genomes (including long-branched taxa). The data set was partitioned by gene, trimmed with BMGE, and analyzed in IQ-TREE 2 with the LG + C60 + G + F mixed model. Bootstrap support values are presented at each node. [file 12862_2020_1728_MOESM3_ESM.pdf]

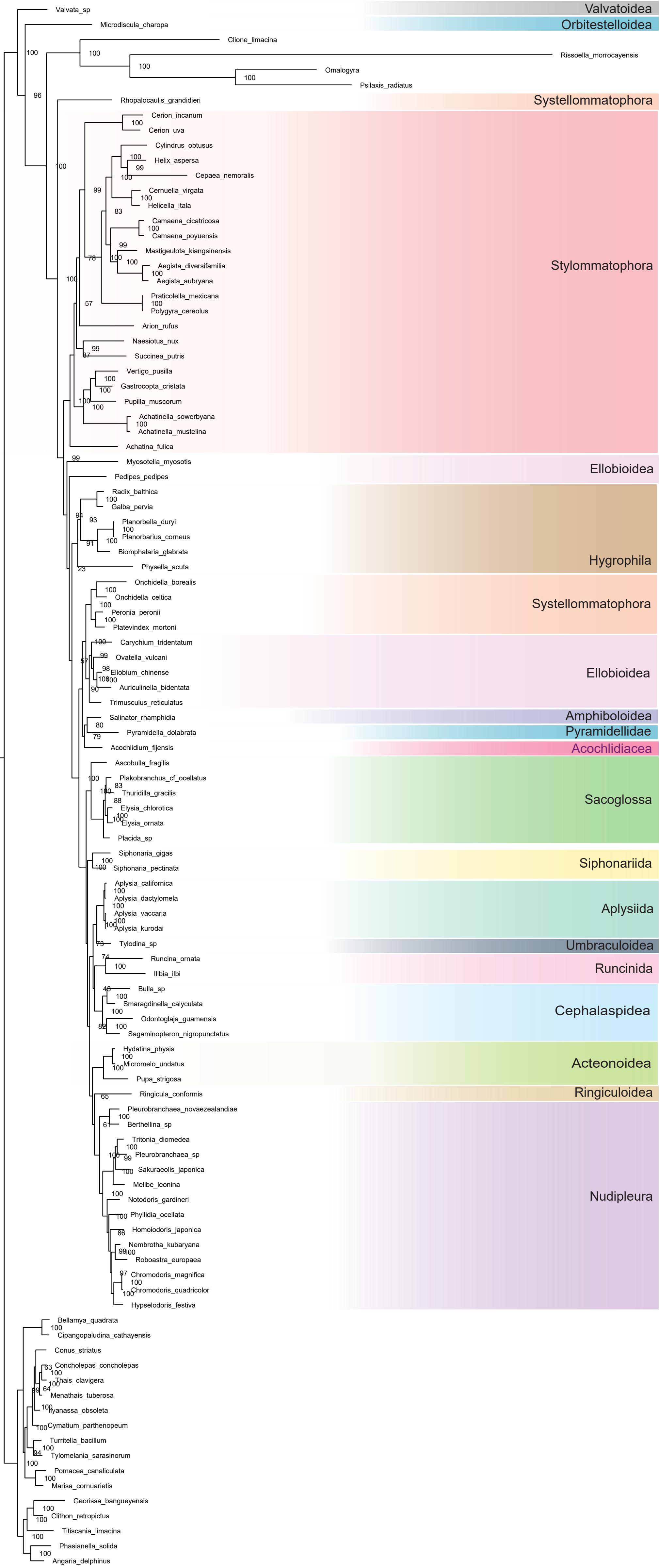

Supplement: Supplementary file 4 — Additional file 4: Figure S3. Maximum likelihood phylogeny of heterobranch gastropods based on the full set of available heterobranch mitochondrial genomes (including long-branched taxa). The data set was partitioned by gene, trimmed with BMGE, and greedy Lanfear clustering was applied in IQ-TREE 2 to determine the optimal partitioning scheme. Five partitions with independent models were applied. Bootstrap support values are presented at each node. [file 12862_2020_1728_MOESM4_ESM.pdf]

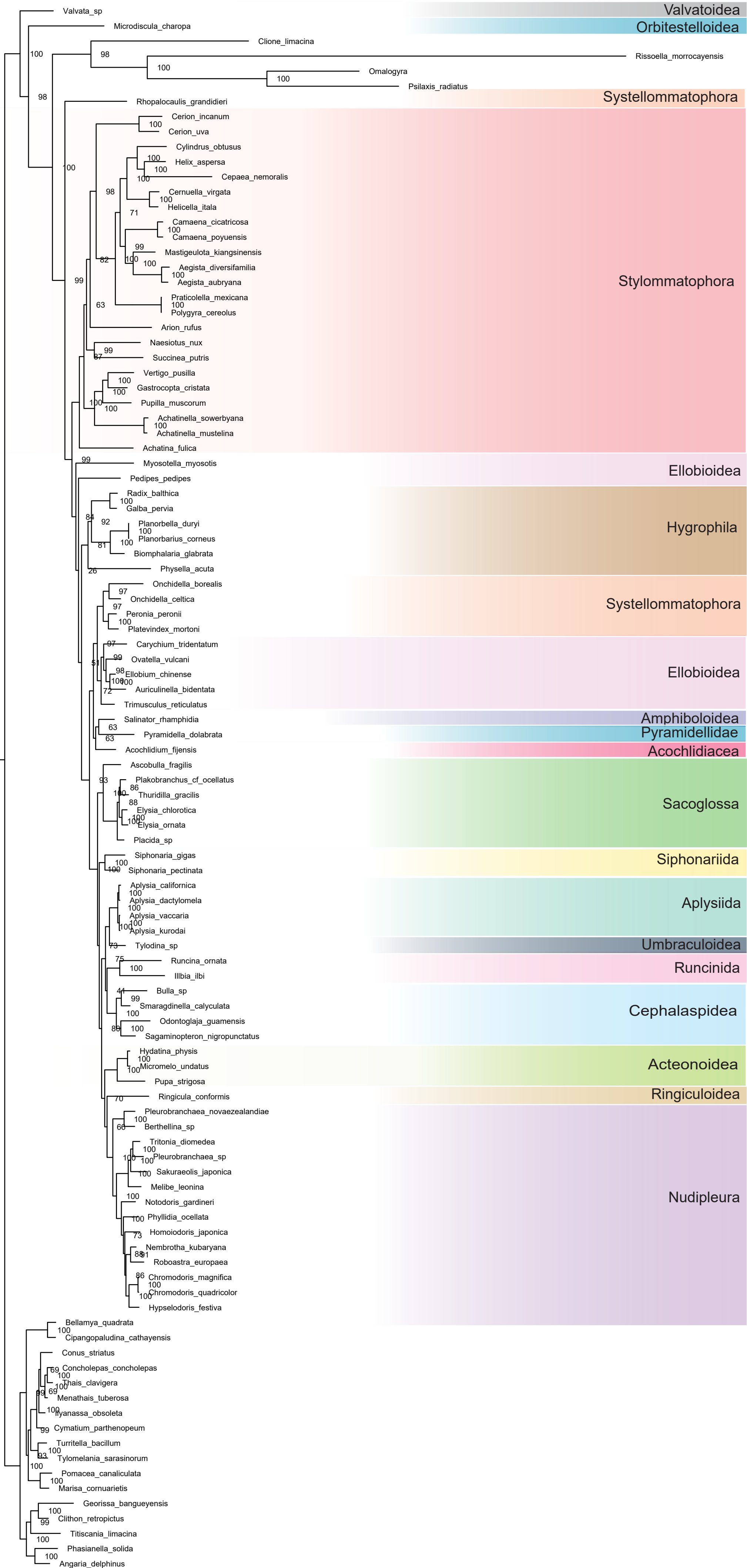

Supplement: Supplementary file 5 — Additional file 5: Figure S4. Maximum likelihood phylogeny of heterobranch gastropods based on the full set of available heterobranch mitochondrial genomes (including long-branched taxa). The data set was partitioned by gene, trimmed with BMGE, and each partition was allowed to select its own optimal model via ModelFinder implemented in IQ-TREE 2. The analysis was run with the –GENESITE correction to facilitate resampling first within partition and then within sites. Bootstrap support values are presented at each node. [file 12862_2020_1728_MOESM5_ESM.pdf]

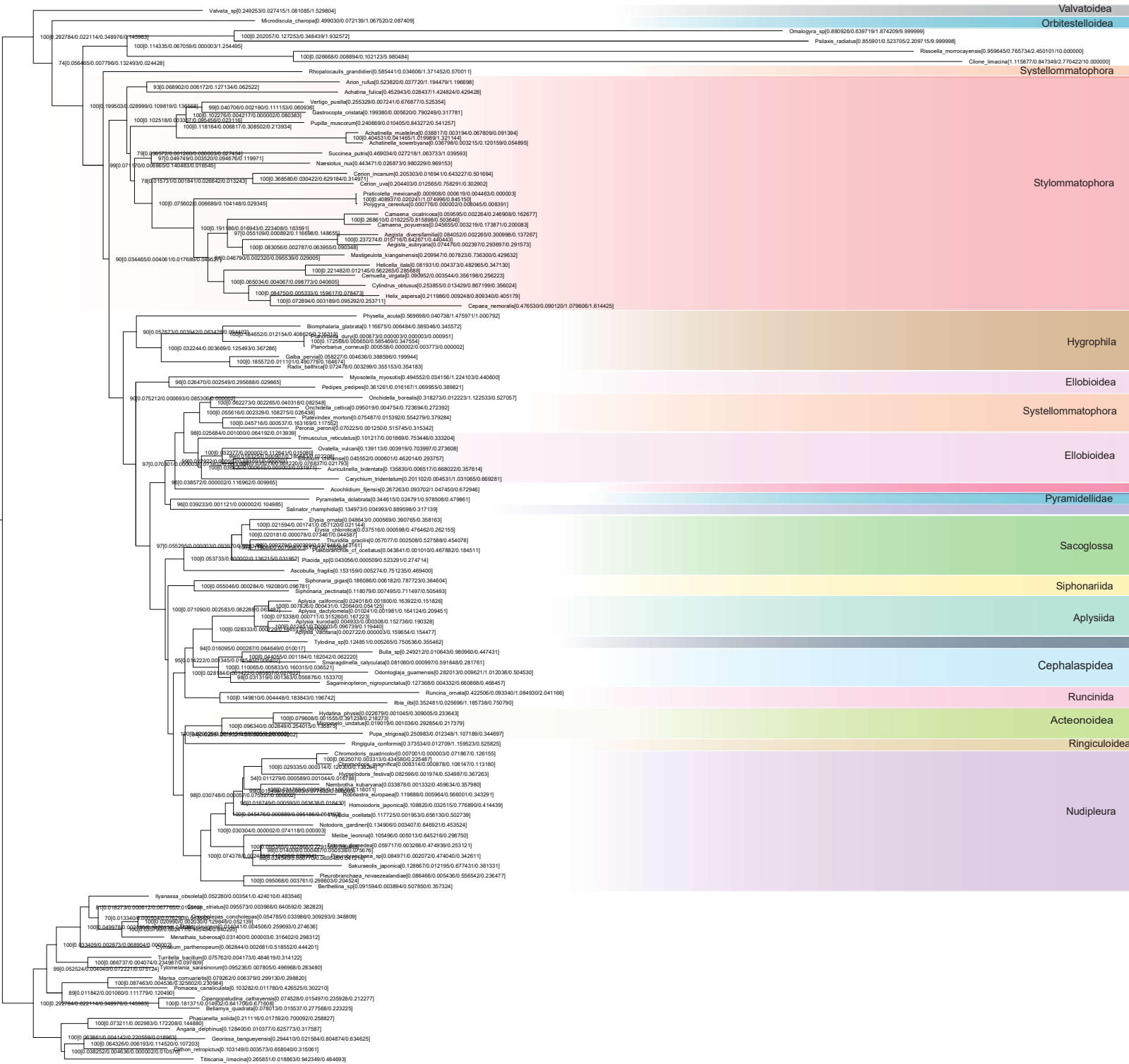

Supplement: Supplementary file 6 — Additional file 6: Figure S5. Maximum likelihood phylogeny of heterobranch gastropods based on the full set of available heterobranch mitochondrial genomes (including long-branched taxa). The data set was trimmed with BMGE, concatenated into a supermatrix, and analyzed with an edge-unlinked model to better account for heterotachy (GHOST). The analysis was run in IQ-TREE 2 with the –GENESITE correction to facilitate resampling first within partition and then within sites. Bootstrap support values are presented at each node. [file 12862_2020_1728_MOESM6_ESM.pdf]

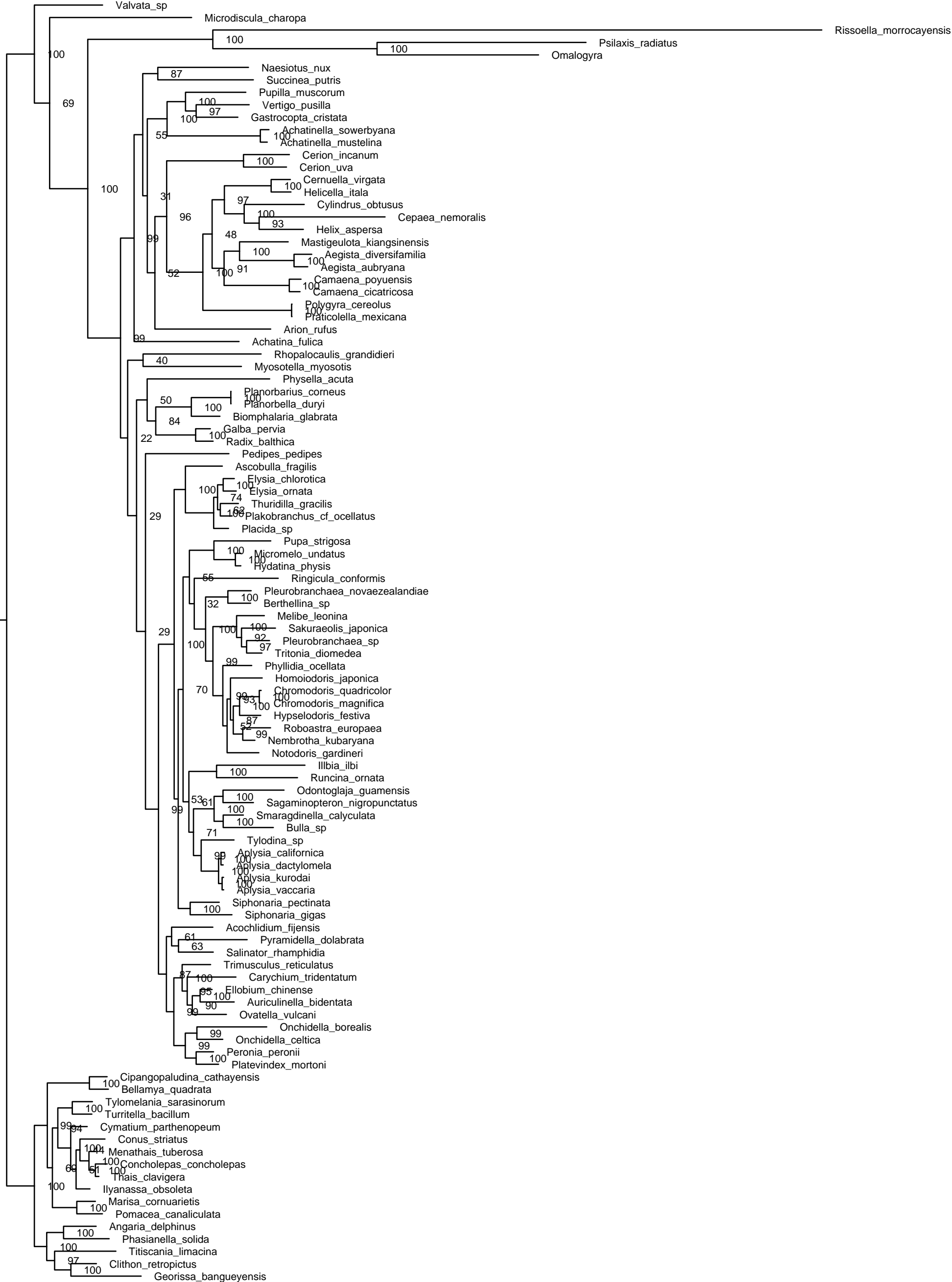

Supplement: Supplementary file 7 — Additional file 7: Figure S6. Maximum likelihood phylogeny of heterobranch gastropods based on the full set of available heterobranch mitochondrial genomes (including long-branched taxa) except for C. limacina. The data set was partitioned, trimmed with TrimAL with default settings, and concatenated into a supermatrix, and run in RAxML with -PROTGAMMAAUTO setting to select the best-fitting model. [file 12862_2020_1728_MOESM7_ESM.pdf]

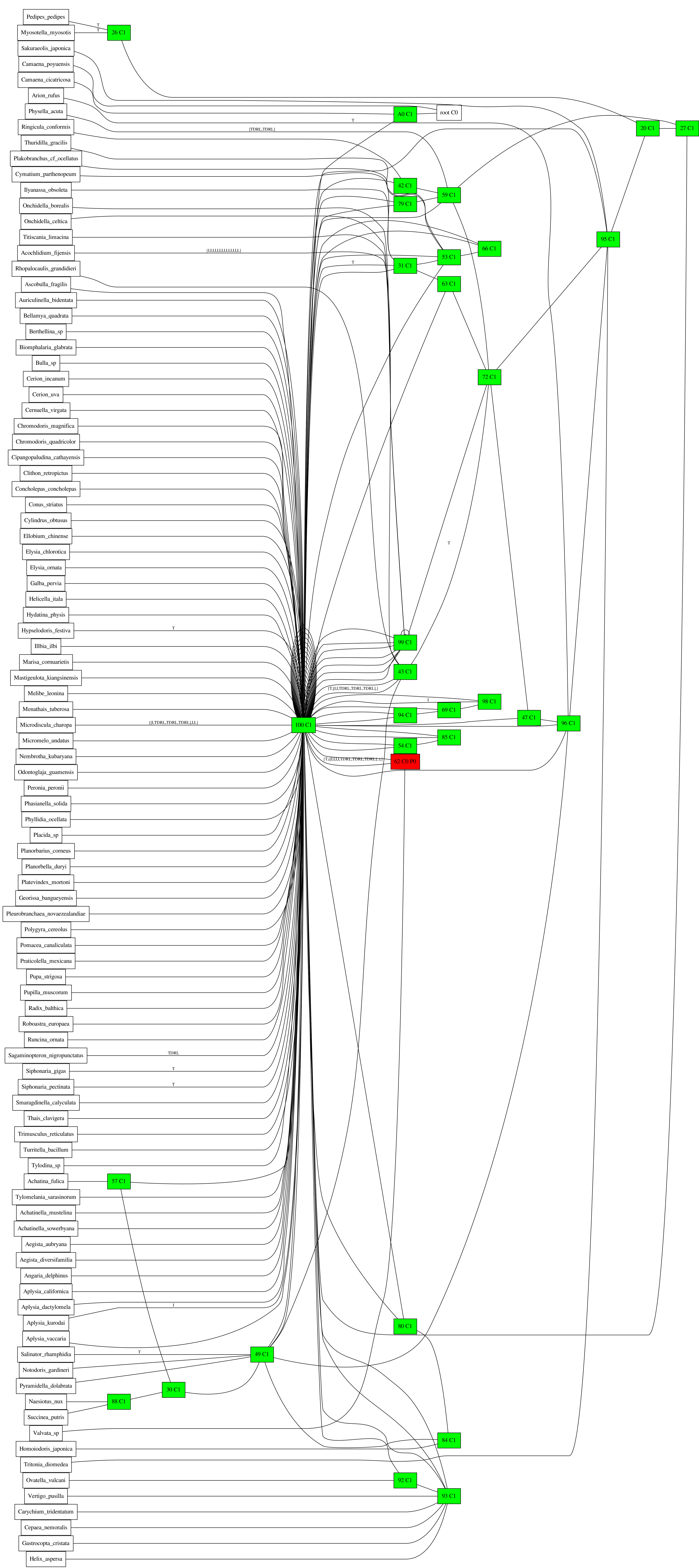

Supplement: Supplementary file 8 — Additional file 8: Figure S7. TreeRex output of a rearrangement analysis highlighting the multiple inversions and transpositions across heterobranch mitochondrial genomes. Rearrangements shown on branches are delineated as T for transposition and TDL for tandem-duplication-random-loss events. Nodes colored green as consistently reconstructed, red reconstructed with the fallback method (where P0 indicates the chosen solution is not better than other possible solutions). [file 12862_2020_1728_MOESM8_ESM.pdf]

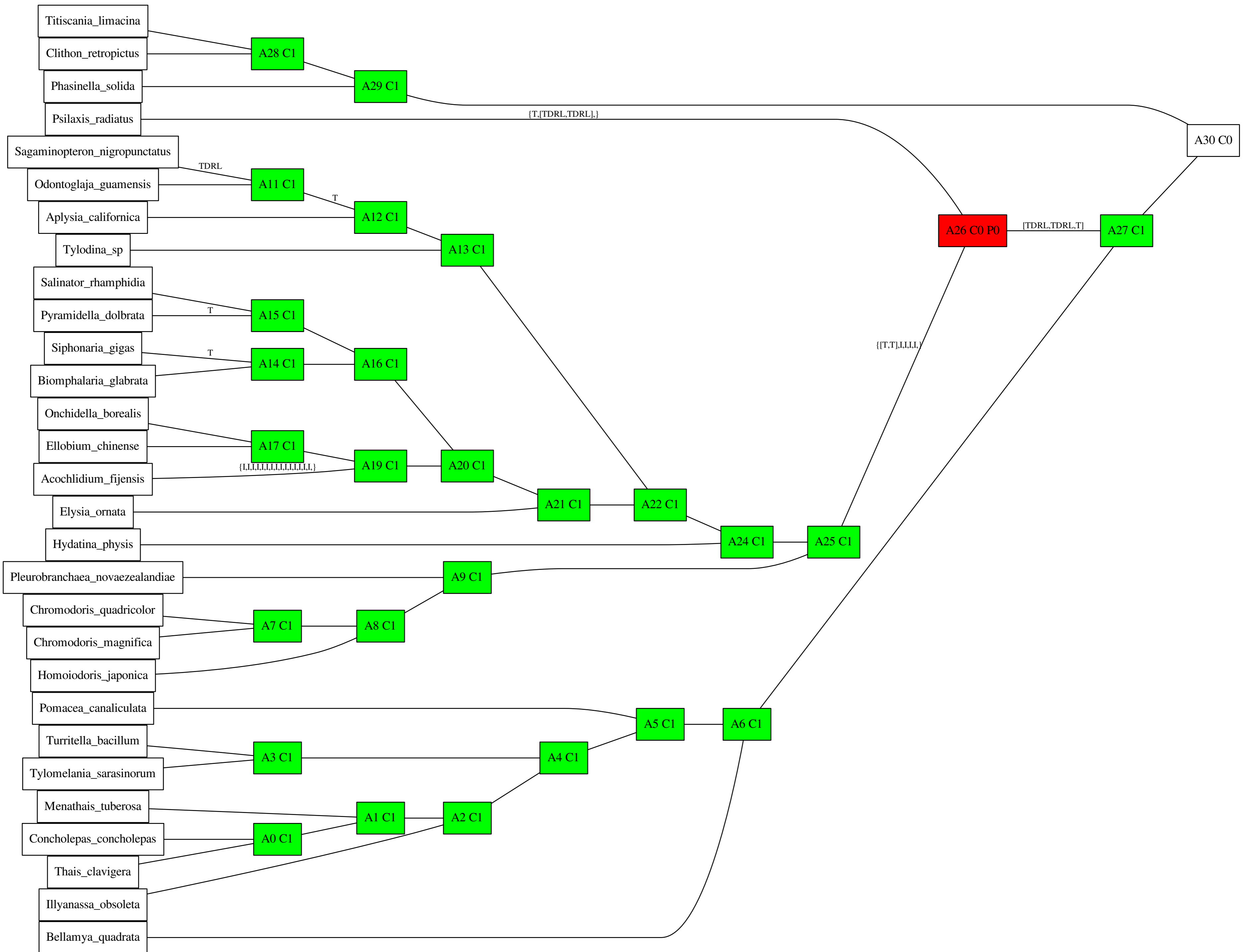

Supplement: Supplementary file 11 — Additional file 11: Table S3. Mitochondrial gene orders in all taxa from the present study, including outgroups, with < and > indicating directionality and orange boxes indicating possible locations of gene rearrangements. [file 12862_2020_1728_MOESM11_ESM.pdf]
