# Supplementary material for: Assessment of mitochondrial genomes for heterobranch gastropod phylogenetics
Source: BMC Ecol Evol. 2021 Jan 21;21:6. doi: 10.1186/s12862-020-01728-y (PMC7853304; doi:10.1186/s12862-020-01728-y)
Supplement: Supplementary file 9 — Additional file 9: Table S2. RogueNaRok leaf instability indices, run with the tree from Figure 1. Lsi_42_max represents the maximum leaf instability across four possible quartets, lsi_42_ent the entropy between the two most different quartets, and Lsi_42_dif the leaf stability differences between the two most common quartets. [file 12862_2020_1728_MOESM9_ESM.docx]

Supplemental Table 2: RogueNaRok leaf instability indices, run with tree from Figure 1. Lsi_42_max represents maximum leaf instability across four possible quartets, lsi_42_ent the entropy between the two most different quartets, and Lsi_42_dif the leaf stability difference between the two most common quartets.

| **Name** | **rnr_mr_1_sup** | **lsi_42_max** | **lsi_42_ent** | **lsi_42_dif** |
| --- | --- | --- | --- | --- |
| Rhopalocaulis_grandidieri | 0.336 | 0.631006 | 0.631006 | 0.676689 |
| Physella_acuta | 0.808 | 0.685023 | 0.685023 | 0.686447 |
| Pedipes_pedipes | 0.588 | 0.669415 | 0.669415 | 0.699763 |
| Myosotella_myosotis | 0.132 | 0.674525 | 0.674525 | 0.708393 |
| Onchidella_borealis | 1.732 | 0.714321 | 0.714321 | 0.739932 |
| Siphonaria_gigas |  | 0.75147 | 0.75147 | 0.79992 |
| Siphonaria_pectinata |  | 0.75147 | 0.75147 | 0.79992 |
| Radix_balthica |  | 0.785551 | 0.785551 | 0.81176 |
| Galba_pervia |  | 0.785551 | 0.785551 | 0.81176 |
| Biomphalaria_glabrata |  | 0.787779 | 0.787779 | 0.812827 |
| Planorbarius_corneus |  | 0.787779 | 0.787779 | 0.812827 |
| Planorbella_duryi |  | 0.787779 | 0.787779 | 0.812827 |
| Salinator_rhamphidia |  | 0.770057 | 0.770057 | 0.819207 |
| Pyramidella_dolabrata |  | 0.770057 | 0.770057 | 0.819207 |
| Runcina_ornata | 0.244 | 0.746865 | 0.746865 | 0.824778 |
| Bellamya_quadrata |  | 0.795556 | 0.795556 | 0.829473 |
| Cipangopaludina_cathayensis |  | 0.795556 | 0.795556 | 0.829473 |
| Pomacea_canaliculata |  | 0.799725 | 0.799725 | 0.8317 |
| Marisa_cornuarietis |  | 0.799725 | 0.799725 | 0.8317 |
| Ilyanassa_obsoleta |  | 0.801733 | 0.801733 | 0.832602 |
| Turritella_bacillum |  | 0.801505 | 0.801505 | 0.832633 |
| Tylomelania_sarasinorum |  | 0.801505 | 0.801505 | 0.832633 |
| Cymatium_parthenopeum |  | 0.801932 | 0.801932 | 0.832743 |
| Conus_striatus |  | 0.802667 | 0.802667 | 0.83332 |
| Concholepas_concholepas |  | 0.803576 | 0.803576 | 0.83392 |
| Menathais_tuberosa |  | 0.803576 | 0.803576 | 0.83392 |
| Thais_clavigera |  | 0.803576 | 0.803576 | 0.83392 |
| Titiscania_limacina_maybe |  | 0.803874 | 0.803874 | 0.834282 |
| Phasianella_solida |  | 0.804352 | 0.804352 | 0.834467 |
| Angaria_delphinus |  | 0.804352 | 0.804352 | 0.834467 |
| Georissa_bangueyensis_maybe |  | 0.804352 | 0.804352 | 0.834467 |
| Clithon_retropictus |  | 0.804352 | 0.804352 | 0.834467 |
| Valvata_sp |  | 0.808209 | 0.808209 | 0.836806 |
| Acochlidium_fijensis |  | 0.808785 | 0.808785 | 0.843698 |
| Naesiotus_nux |  | 0.823613 | 0.823613 | 0.848038 |
| Arion_rufus | 0.12 | 0.821977 | 0.821977 | 0.848156 |
| Placida_sp |  | 0.815352 | 0.815352 | 0.848464 |
| Plakobranchus_cf_ocellatus |  | 0.815505 | 0.815505 | 0.848585 |
| Thuridilla_gracilis |  | 0.815642 | 0.815642 | 0.848727 |
| Succinea_putris |  | 0.822166 | 0.822166 | 0.848739 |
| Elysia_chlorotica |  | 0.816081 | 0.816081 | 0.849039 |
| Elysia_ornata |  | 0.816081 | 0.816081 | 0.849039 |
| Ascobulla_fragilis |  | 0.816973 | 0.816973 | 0.849681 |
| Onchidella_celtica |  | 0.827035 | 0.827035 | 0.851952 |
| Platevindex_mortoni |  | 0.82741 | 0.82741 | 0.852369 |
| Peronia_peronii |  | 0.827414 | 0.827414 | 0.852377 |
| Trimusculus_reticulatus |  | 0.827245 | 0.827245 | 0.852646 |
| Carychium_tridentatum |  | 0.829307 | 0.829307 | 0.854278 |
| Ovatella_vulcani |  | 0.829392 | 0.829392 | 0.854307 |
| Ellobium_chinense |  | 0.82967 | 0.82967 | 0.854517 |
| Auriculinella_bidentata |  | 0.82967 | 0.82967 | 0.854517 |
| Odontoglaja_guamensis |  | 0.824107 | 0.824107 | 0.861619 |
| Sagaminopteron_nigropunctatus |  | 0.824107 | 0.824107 | 0.861619 |
| Bulla_sp |  | 0.824119 | 0.824119 | 0.861624 |
| Smaragdinella_calyculata |  | 0.824135 | 0.824135 | 0.861627 |
| Aplysia_californica |  | 0.824357 | 0.824357 | 0.861684 |
| Aplysia_vaccaria |  | 0.824357 | 0.824357 | 0.861684 |
| Aplysia_dactylomela |  | 0.824357 | 0.824357 | 0.861684 |
| Aplysia_kurodai |  | 0.824357 | 0.824357 | 0.861684 |
| Achatina_fulica |  | 0.834135 | 0.834135 | 0.861932 |
| Micromelo_undatus |  | 0.825242 | 0.825242 | 0.867017 |
| Pupa_strigosa |  | 0.825242 | 0.825242 | 0.867017 |
| Hydatina_physis |  | 0.825242 | 0.825242 | 0.867017 |
| Polygyra_cereolus |  | 0.858638 | 0.858638 | 0.873087 |
| Praticolella_mexicana |  | 0.858638 | 0.858638 | 0.873087 |
| Gastrocopta_cristata |  | 0.853566 | 0.853566 | 0.874173 |
| Vertigo_pusilla |  | 0.853566 | 0.853566 | 0.874173 |
| Pupilla_muscorum |  | 0.853566 | 0.853566 | 0.874173 |
| Achatinella_sowerbyana |  | 0.853995 | 0.853995 | 0.874412 |
| Achatinella_mustelina |  | 0.853995 | 0.853995 | 0.874412 |
| Notodoris_gardineri | 0.064 | 0.846804 | 0.846804 | 0.878214 |
| Homoiodoris_japonica | 0.028 | 0.847039 | 0.847039 | 0.878408 |
| Roboastra_europaea |  | 0.849449 | 0.849449 | 0.880003 |
| Nembrotha_kubaryana |  | 0.849983 | 0.849983 | 0.880414 |
| Phyllidia_ocellata |  | 0.850154 | 0.850154 | 0.881595 |
| Camaena_poyuensis |  | 0.864268 | 0.864268 | 0.881794 |
| Camaena_cicatricosa |  | 0.864268 | 0.864268 | 0.881794 |
| Hypselodoris_festiva |  | 0.852096 | 0.852096 | 0.882071 |
| Chromodoris_quadricolor |  | 0.852157 | 0.852157 | 0.882091 |
| Chromodoris_magnifica |  | 0.852157 | 0.852157 | 0.882091 |
| Mastigeulota_kiangsinensis |  | 0.864794 | 0.864794 | 0.882194 |
| Aegista_diversifamilia |  | 0.86491 | 0.86491 | 0.882204 |
| Aegista_aubryana |  | 0.86491 | 0.86491 | 0.882204 |
| Cernuella_virgata |  | 0.864716 | 0.864716 | 0.882244 |
| Helicella_itala |  | 0.864716 | 0.864716 | 0.882244 |
| Cepaea_nemoralis |  | 0.865115 | 0.865115 | 0.882332 |
| Cylindrus_obtusus |  | 0.865115 | 0.865115 | 0.882332 |
| Helix_aspersa |  | 0.865115 | 0.865115 | 0.882332 |
| Sakuraeolis_japonica |  | 0.853645 | 0.853645 | 0.883903 |
| Melibe_leonina |  | 0.853712 | 0.853712 | 0.883924 |
| Tritonia_diomedea |  | 0.853715 | 0.853715 | 0.883953 |
| Pleurobranchaea_sp |  | 0.853693 | 0.853693 | 0.883953 |
| Berthellina_sp |  | 0.854306 | 0.854306 | 0.884149 |
| Pleurobranchaea_novaezealandiae |  | 0.854306 | 0.854306 | 0.884149 |
| Cerion_incanum |  | 0.867983 | 0.867983 | 0.888009 |
| Cerion_uva |  | 0.867983 | 0.867983 | 0.888009 |
